# Gallic Acid Inhibits Lipid Accumulation via AMPK Pathway and Suppresses Apoptosis and Macrophage-Mediated Inflammation in Hepatocytes

**DOI:** 10.3390/nu12051479

**Published:** 2020-05-20

**Authors:** Miori Tanaka, Akari Sato, Yoshimi Kishimoto, Hideaki Mabashi-Asazuma, Kazuo Kondo, Kaoruko Iida

**Affiliations:** 1 Department of Food and Nutritional Sciences, Graduate School of Humanities and Sciences, Ochanomizu University, 2-1-1 Otsuka, Bunkyo-ku, Tokyo 112-8610, Japan; mt207326@nodai.ac.jp (M.T.); g1840530@edu.cc.ocha.ac.jp (A.S.); mabashi.hideaki@ocha.ac.jp (H.M.-A.); 2 Department of Nutritional Science and Food Safety, Faculty of Applied Bioscience, Tokyo University of Agriculture, 1-1-1 Sakuragaoka, Setagaya-ku, Tokyo 156-8502, Japan; 3 Endowed Research Department “Food for Health”, Ochanomizu University, 2-1-1 Otsuka, Bunkyo-ku, Tokyo 112-8610, Japan; kishimoto.yoshimi@ocha.ac.jp (Y.K.); kondo002@toyo.jp (K.K.); 4 Institute for Human Life Innovation, Ochanomizu University, 2-1-1 Otsuka, Bunkyo-ku, Tokyo 112-8610, Japan; 5 Department of Nutrition and Health Sciences, Toyo University, 1-1-1 Izumino, Itakura-machi, Ora-gun, Gunma 374-0193, Japan

**Keywords:** gallic acid, polyphenol, nonalcoholic steatohepatitis, hepatocyte, macrophage

## Abstract

Nonalcoholic fatty liver disease (NAFLD) is one of the most common causes of chronic liver disease, sometimes ranges from simple steatosis to nonalcoholic steatohepatitis (NASH). Various hits including excessive hepatic steatosis, oxidative stress, apoptosis, and inflammation, contribute to NASH development. Gallic acid (GA), a natural polyphenol, was reported to exert a protective effect on hepatic steatosis in animal models, but the precise molecular mechanisms remain unclear. Here, we examined the effect of GA on hepatic lipid accumulation, apoptosis, and inflammatory response caused by hepatocyte–macrophage crosstalk. We demonstrated that GA attenuated palmitic acid (PA)-induced fat accumulation via the activation of AMP-activated protein kinase (AMPK) in HepG2 cells. GA also ameliorated cell viability and suppressed apoptosis-related gene expression and caspase 3/7 activity induced by PA and H_2_O_2_. In a co-culture of lipid-laden Hepa 1-6 hepatocytes and RAW 264 macrophages, GA reduced inflammatory mediator expression and induced antioxidant enzyme expression. These results indicate that GA suppresses hepatic lipid accumulation, apoptosis, and inflammation caused by the interaction between hepatocytes and macrophages. The potential effects of GA observed in our study could be effective in preventing NASH and its complications.

## 1. Introduction

Nonalcoholic fatty liver disease (NAFLD), which is the hepatic manifestation of metabolic syndrome, is one of the most common causes of chronic liver diseases such as cirrhosis and hepatocellular carcinoma [1]. The major risk factors of NAFLD include obesity, type 2 diabetes, and hyperlipidemia [2], and the estimated prevalence of NAFLD worldwide is approximately 25% [3]. NAFLD is generally defined as fatty liver with intrahepatic triglyceride exceeding 5% of the whole weight, which can range from simple steatosis to nonalcoholic steatohepatitis (NASH) with or without cirrhosis development [4]. Although NAFLD increases the risk of cardiovascular diseases as well as chronic liver diseases [5], there is no established therapeutic approach against NAFLD and progressive NASH.

The pathological features of NASH include steatosis, hepatocyte apoptosis, inflammation, and fibrosis. The precise mechanisms involved in the pathogenesis of NASH have not been fully elucidated, but one of the well-accepted mechanisms is the “multiple parallel hits” hypothesis [6]. In this proposal, various parallel hits including excessive hepatic lipid accumulation, oxidative stress, lipotoxicity, and inflammatory mediators, may contribute to NASH development. Hepatic steatosis reflects disordered lipid metabolism when lipid input exceeds the output. Uptake of circulating free fatty acids (FFAs) and *de novo* lipogenesis are major biological lipid sources for hepatocytes [7]. Previous studies suggested that excessive hepatic lipid accumulation induces oxidative stress and subsequent hepatocyte apoptosis, eventually leading to liver fibrosis [8,9].

Chronic inflammation is an important pathogenic factor in metabolic diseases, and macrophages regulate inflammation by producing proinflammatory cytokines including tumor necrosis factor-alpha (TNF-α) and interleukin-1 beta (IL-1β) [10]. In obese adipose tissue, macrophages infiltrate into hypertrophied adipocytes and form a unique histological structure called a crown-like structure (CLS), where adipocyte–macrophage interaction occurs [11]. FFAs from adipocytes increase inflammatory and fibrogenic genes expression in macrophages, thereby inducing insulin resistance, adipose tissue inflammation, fibrosis, and ectopic lipid accumulation [12,13]. Additionally, a recent report revealed that hepatic CLS (hCLS), a CLS-like structure where macrophages surround hepatocytes with large lipid droplets in the liver, is critically associated with hepatic inflammation and fibrosis in NASH mice and patients [14]. Therefore, ameliorating chronic inflammation caused by hepatocyte–macrophage interaction could be important for therapeutic strategies against NASH.

Gallic acid (GA) is a natural polyphenol and found in many plants such as fruits and nuts. GA has been reported to show antioxidant [15] and anti-inflammatory [16] properties in cell-free assays and in lipopolysaccharide-stimulated macrophages. In addition, GA exerted a hypoglycemic effect and improved hepatic carbohydrate metabolism in rats fed high-fructose diets [17]. Several reports have suggested that GA also ameliorated hepatic steatosis and inflammation in high-fat diet-induced and in methionine/choline deficient diet-induced NASH animal models [18,19,20], but there is little information about the precise molecular mechanisms of GA. In addition, we recently demonstrated that GA suppressed adverse interaction between adipocytes and macrophages, thereby improving obesity-induced adipose tissue inflammation and metabolic disorders in vitro and in mice fed high-fat high-sucrose diets [21]. However, whether GA attenuates chronic inflammation in the liver as well as in adipose tissue remains unclear. Thus, the aim of this study was to examine the protective effect of GA on lipid accumulation, apoptosis, and underlying molecular mechanisms in hepatocytes. We also investigated whether GA inhibits inflammatory response in a co-culture system of lipid-laden hepatocytes and macrophages as an in vitro model of hepatic inflammation.

## 2. Materials and Methods

### 2.1. Reagents

GA, palmitic acid (PA), Oil Red O, compound C, and 3-(4,5-dimethylthiazol-2-yl)-2,5-diphenyltetrazoliumbromide (MTT) were purchased from Sigma-Aldrich (St Louis, MO, USA). Oleic acid (OA) and Dulbecco’s modified eagle medium (DMEM) were acquired from Nacalai Tesque (Kyoto, Japan). Fetal bovine serum (FBS) and penicillin/streptomycin were obtained from Gibco (Life Technologies, Carlsbad, CA, USA).

### 2.2. Cell Culture and Treatment

The human hepatoma cell line HepG2, murine hepatoma cell line Hepa 1-6, and murine macrophage cell line RAW 264 (RIKEN Cell Bank, Ibaraki, Japan) were cultured in DMEM supplemented with 10% FBS, 100 U/mL penicillin and 100 µg/mL streptomycin at 37 °C and 5% CO_2_. To prepare fatty acid (FA) solutions, PA and OA were dissolved in 100 mM NaOH for 15 min at 70 °C, respectively, and 100 mM FA solutions were then mixed with prewarmed FA-free BSA (10% in DMEM) to yield 8 mM PA or OA stock solution. The solutions were incubated for 15 min at 55 °C and stored at −20 °C until use.

### 2.3. Cell Viability

MTT assay was used to detect cell viability. HepG2 cells were seeded in 24-well plates at a density of 3.5 × 10^5^ cells/mL and incubated for 48 h. Cells were treated with 50–200 µM GA for 24 h. In apoptosis assay, HepG2 cells were pretreated with 50–200 µM GA for 24 h, then PA (400 µM) and H_2_O_2_ (400 and 800 µM) in fresh medium were added and incubated for 24 h. After treatment, cells were incubated with fresh media containing MTT (0.5 mg/mL) for 3 h. The solution was then carefully removed, and dimethyl sulfoxide was added to dissolve the resulting formazan crystals. The absorbance was measured at 540 nm using a microplate reader (Bio Tek Instruments, Tokyo, Japan).

### 2.4. Oil Red O Staining

HepG2 cells were seeded in 24-well plates at a density of 3.5 × 10^5^ cells/mL and incubated for 48 h. Cells were treated with 400 µM PA and 50–200 µM GA for 24 h. After washing twice with PBS, the cells were fixed with 10% formalin for 10 min, washed twice with PBS, and stained with Oil Red O solution for 35 min. Cells were then washed twice with PBS and photographed under a BZ-X710 fluorescence microscope (20× objective lens, KEYENCE, Osaka, Japan). The extent of steatosis was quantified by isolating cytoplasmic lipids with isopropanol and measuring the absorbance at 500 nm using a microplate reader.

### 2.5. Real-Time PCR

HepG2 cells were seeded in 12-well plates at a density of 2.5 × 10^5^ cells/mL and incubated for 72 h. For lipid accumulation, cells were treated with 400 µM PA and 50–200 µM GA for 24 h. For apoptotic induction, cells were pretreated with 200 µM GA for 24 h, then the medium was replaced with the fresh medium containing PA (400 µM) and H_2_O_2_ (400 µM) and incubated for 12 h. Total RNA was extracted using RNAiso Plus (Takara Bio, Shiga, Japan), in accordance with the manufacturer’s instructions. We reverse-transcribed first-strand complementary DNA from 2 µg of total RNA using a High Capacity cDNA Reverse Transcription Kit (Applied Biosystems, Foster City, CA, USA). Real-time PCR was performed on a StepOnePlus Real-Time PCR System (Applied Biosystems) using Power SYBR Green PCR mix (Applied Biosystems). The results are expressed as the copy number ratio of the target mRNA to GAPDH mRNA. Primers of genes encoding cluster of differentiation 36 (CD36) (*CD36*), fatty acid transport protein 2 (FATP2) (*SLC27A2*), acetyl-CoA carboxylase alpha (ACCα) (*ACACA*), sterol regulatory element-binding protein-1c (SREBP-1c) (*SREBF1*), liver X receptor-alpha (LXRα) (*NR1H3*), Bcl-2-associated X protein (Bax) (*BAX*), B-cell lymphoma 2 (Bcl-2) (*BCL2*), activating transcription factor 3 (ATF3) (*ATF3*), TNF-α (*Tnf*), IL-1β (*Il1b*), monocyte chemoattractant protein-1 (MCP-1) (*Ccl2*), inducible nitric oxide synthase (iNOS) (*Nos2*), heme oxygenase-1 (HO-1) (*Hmox1*), and catalase (*Cat*) were obtained from Sigma-Aldrich. Primer sequences are listed in Table 1.

### 2.6. Western Blot Analysis

HepG2 cells were seeded in 6-well plates at a density of 3.1 × 10^5^ cells/mL and incubated for 72 h. Cells were treated with 400 µM PA and 50–200 µM GA for 24 h. Total protein was extracted using RIPA buffer (Nacalai Tesque, Kyoto, Japan). Equal amounts of cellular proteins were electrophoresed on 10% sodium dodecyl sulfate-polyacrylamide gels and transferred to Hybond-P membranes (GE Healthcare, Little Chalfont, UK). Membranes were blocked with 5% BSA, and incubated with primary antibodies against CD36, SREBP-1c, β-actin (Santa Cruz Biotechnology, Dallas, TX, USA), and AMP-activated protein kinase (AMPK) (Cell Signaling Technology, Danvers, MA, USA). After washing with TBS-T, membranes were incubated with peroxidase-conjugated secondary antibodies: anti-rabbit and anti-mouse (Cell Signaling Technology). Chemiluminescent detection of specific proteins was developed with ECL Select Western Blotting Detection Reagent (GE Healthcare). All signals were detected using an ImageQuant LAS 4000 system (GE Healthcare). The optical density of each band was quantified by ImageJ software (National Institutes of Health, Bethesda, MD, USA).

### 2.7. Caspase 3/7 Activity

We tested caspase 3/7 activity by using a Caspase-Glo 3/7 Assay System (Promega, Madison, WI, USA). HepG2 cells were seeded in 96-well plates at a density of 3.0 × 10^5^ cells/mL and incubated for 48 h. Cells were pretreated with 200 µM GA for 24 h, then PA (400 µM) and H_2_O_2_ (400 µM) in fresh medium were added and incubated for 24 h. After treatment, 100 µL of Caspase-Glo 3/7 Reagent was added to each well, and the plate was then placed on a shaker for 30 s and incubated for 30 min at room temperature. Luminescence was determined using an EnSpire multimode plate reader (PerkinElmer, Waltham, MA, USA).

### 2.8. Co-Culture of Hepatocytes and Macrophages

To use hepatocyte and macrophage cell lines originated from the same animal species, Hepa 1-6 cells instead of HepG2 cells were used in a co-culture system. Hepa 1-6 cells were seeded in 12-well plates at a density of 1.2 × 10^5^ cells/mL and incubated for 72 h. Cells were treated with 400 µM FA (PA:OA = 1:2) for 24 h, then the medium containing FA was removed and RAW 264 macrophages (1.0 × 10^5^ cells/well) were placed onto hepatocytes in serum-free medium with or without 25 µM GA. Cells were incubated for 24 h and harvested. As a control culture, lipid-laden Hepa 1-6 cells and RAW 264 macrophages, the numbers of which were equal to those in the co-culture, were cultured separately and mixed after harvest. Total RNA was extracted from the samples and subjected to real-time PCR as described in Section 2.5.

### 2.9. Statistical Analysis

Comparisons between treatment groups were performed using a one-way analysis of variance (ANOVA) followed by Tukey’s post hoc test. Differences were considered statistically significant when *p* < 0.05. The GraphPad Prism 5 software package (GraphPad Software, La Jolla, CA, USA) was used to perform statistical analyses.

## 3. Results

### 3.1. GA Inhibited Lipid Accumulation in HepG2 Cells

**Figure 1:**
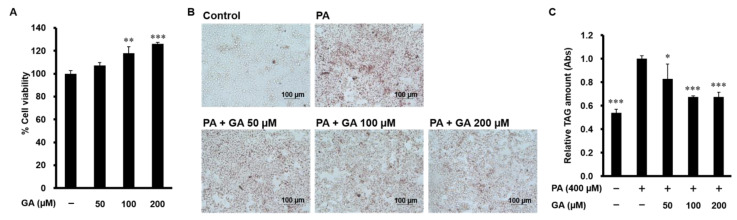
Effect of gallic acid (GA) on lipid accumulation in palmitic acid (PA)-stimulated HepG2 cells. (**A**) HepG2 cells were treated with 50–200 µM GA for 24 h. Cell viability was determined by MTT assay. Data represent mean ± SD, *n* = 3. (** *p* < 0.01, *** *p* < 0.001 compared to control group.) (**B**,**C**) HepG2 cells were treated with 400 µM PA in the absence or presence of 50–200 µM GA for 24 h. Intracellular triglyceride levels were measured by Oil Red O staining. (**B**) Representative images are shown. (**C**) The extent of steatosis was quantified by the extract in absolute isopropanol. Data represent mean ± SD, *n* = 3. (* *p* < 0.05, *** *p* < 0.001 compared to PA group.).

We first confirmed that GA (50, 100, and 200 µM) had no cytotoxic effect on HepG2 cells (Figure 1A). To examine the effect of GA on hepatocyte lipid accumulation in HepG2 cells, we performed Oil Red O staining. Visualized and quantitative data showed that lipid deposition was increased in PA-treated cells, which was significantly inhibited by GA treatment (Figure 1B,C).

### 3.2. Effect of GA on Steatosis-Related Gene and Protein Expression in HepG2 Cells

**Figure 2:**
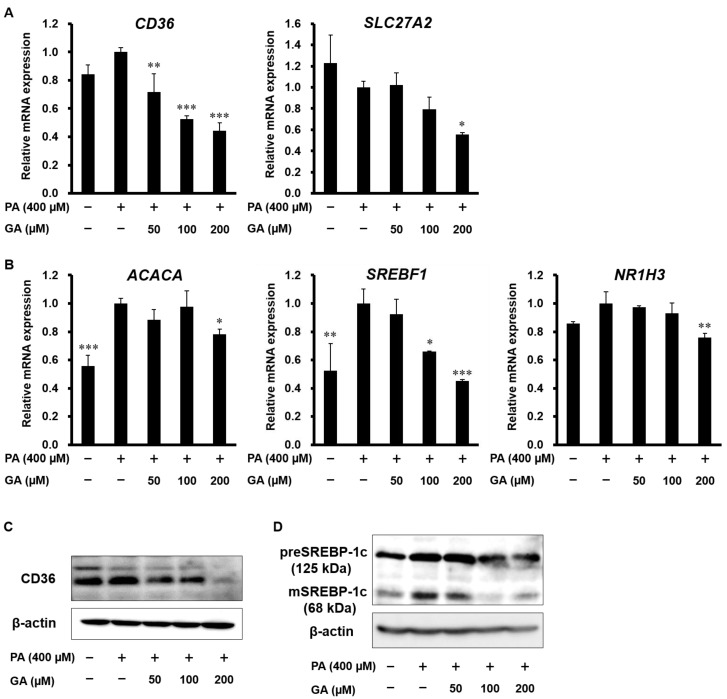
Effect of GA on fatty acid metabolism-related gene and protein expression in PA-stimulated HepG2 cells. (**A**–**D**) HepG2 cells were treated with 400 µM PA in the absence or presence of 50–200 µM GA for 24 h. (**A**) The mRNA levels of *CD36* and *SLC27A2* were determined by real-time PCR. (**B**) The mRNA levels of *ACACA*, *SREBF1*, and *NR1H3* were determined by real-time PCR. (**C**) The protein levels of CD36 were detected by western blotting. (**D**) The protein levels of precursor and mature SREBP-1c were detected by western blotting. Data represent mean ± SD, *n* = 3. (* *p* < 0.05, ** *p* < 0.01, *** *p* < 0.001 compared to PA group.).

To evaluate the regulatory mechanisms of GA on fat accumulation, we investigated whether GA affects fatty acid metabolism-related gene and protein expression in HepG2 cells. As shown in Figure 2A, PA had no effect on the mRNA expression of fatty acid transporters, such as CD36 and FATP2. Treatment with GA significantly downregulated the expression of these genes. SREBP-1c and LXRα are key transcription factors that regulate fatty acid synthesis. PA increased gene expression levels of ACCα and SREBP-1c, while GA showed significant inhibition of ACCα, SREBP-1c, and LXRα expression (Figure 2B). We also examined the protein levels of CD36 and SREBP-1c, which showed large fluctuations in gene expression. GA effectively suppressed the protein expression of CD36 and both precursor and mature SREBP-1c (Figure 2C,D).

### 3.3. Blocking AMPK Signaling Inhibited GA-Mediated Reduction of Lipid Accumulation in HepG2 Cells

**Figure 3:**
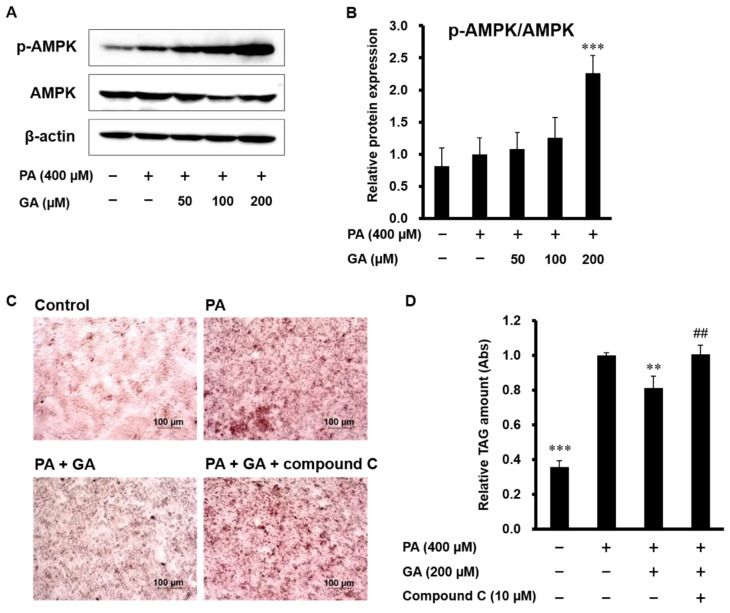
Inhibition of AMPK prevented GA-induced reduction of lipid accumulation in PA-stimulated HepG2 cells. (**A**,**B**) HepG2 cells were treated with 400 µM PA in the absence or presence of 50–200 µM GA for 24 h. AMPK activation was assessed by measuring p-AMPK. (**A**) Representative images are shown. (**B**) The protein levels of p-AMPK were determined and normalized to those of AMPK by ImageJ software. Data represent mean ± SD, *n* = 5. (*** *p* < 0.001 compared to PA group.) (**C**,**D**) HepG2 cells were pretreated with 10 µM compound C for 1 h, followed by treatment with 400 µM PA and 200 µM GA for 24 h. Intracellular triglyceride levels were measured by Oil Red O staining. (**C**) Representative images are shown. (**D**) The extent of steatosis was quantified by the extract in absolute isopropanol. Data represent mean ± SD, *n* = 4. (** *p* < 0.01, *** *p* < 0.001 compared to PA group; ^##^ *p* < 0.01 compared to GA group.).

As AMPK is known to inhibit SREBP-1c and LXRα activities and *de novo* lipogenesis [22,23], we examined the effect of GA on AMPK activation. PA had almost no effect on the phosphorylation of AMPK, but a significant level of AMPK phosphorylation occurred in cells treated with GA (Figure 3A,B). We also confirmed whether AMPK is responsible for GA-induced inhibition of lipid accumulation in HepG2 cells. To this end, cells were pretreated with compound C, an AMPK inhibitor, before GA treatment. As shown in Figure 3C,D, GA significantly reduced fat accumulation, but pretreatment with compound C significantly inhibited the decrease in lipid deposition by GA. These results show that AMPK activation accounts for GA-induced inhibition of lipid accumulation.

### 3.4. GA Attenuated Apoptosis Induced by PA and H_2_O_2_ in HepG2 Cells

**Figure 4:**
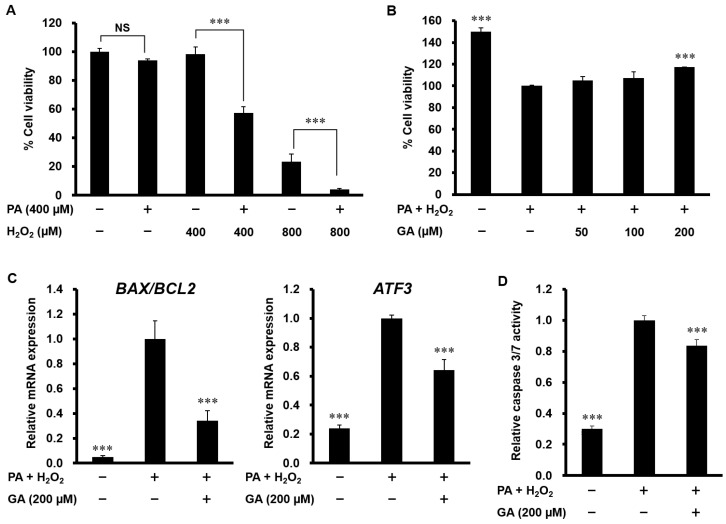
Effect of GA on apoptosis in PA and H_2_O_2_-stimulated HepG2 cells. (**A**) HepG2 cells were treated with 400 µM PA and 400, 800 µM H_2_O_2_ for 24 h. Cell viability was determined by MTT assay. Data represent mean ± SD, *n* = 3. (*** *p* < 0.001; NS, no significant deference.) (**B**) HepG2 cells were pretreated with 50–200 µM GA for 24 h, followed by treatment with 400 µM PA and 400 µM H_2_O_2_ for 24 h. Cell viability was determined by MTT assay. (**C**) HepG2 cells were pretreated with 200 µM GA for 24 h, followed by treatment with 400 µM PA and 400 µM H_2_O_2_ for 12 h. The mRNA levels of *BAX*, *BCL2*, and *ATF3* were determined by real-time PCR. (**D**) HepG2 cells were pretreated with 200 µM GA for 24 h, followed by treatment with 400 µM PA and 400 µM H_2_O_2_ for 24 h. Caspase 3/7 activity was measured using a Caspase-Glo 3/7 Assay System. Data represent mean ± SD, *n* = 3. (*** *p* < 0.001 compared to PA + H_2_O_2_ group.).

Since hepatocyte apoptosis is associated with NASH development, we next analyzed apoptosis in HepG2 cells. MTT assay showed that PA alone or a low concentration of H_2_O_2_ (400 µM) alone had no effect on cell viability; however, H_2_O_2_ (400 and 800 µM) strongly caused cell death in cells co-stimulated with PA (Figure 4A). As shown in Figure 4B, GA (200 µM) significantly ameliorated cell viability in HepG2 cells treated with 400 µM PA and H_2_O_2_. The increase in the ratio of Bax/Bcl-2 induces mitochondrial outer membrane permeabilization and the release of cytochrome c, thereby triggering the apoptosis pathway. As shown in Figure 4C, PA and H_2_O_2_ increased the ratio of Bax/Bcl-2 mRNA expression and the mRNA level of ATF3, a transcription factor that regulates Bax and Bcl-2 expression. Treatment with GA significantly suppressed both of these apoptotic changes. Consistent with these results, an increased level of caspase 3/7 activity was detected in PA and H_2_O_2_-treated cells, which was significantly prevented by GA (Figure 4D).

### 3.5. Effect of GA on Inflammatory Mediator and Antioxidant Enzyme Expression in a Co-Culture of Hepa 1-6 Cells and RAW 264 Macrophages

**Figure 5:**
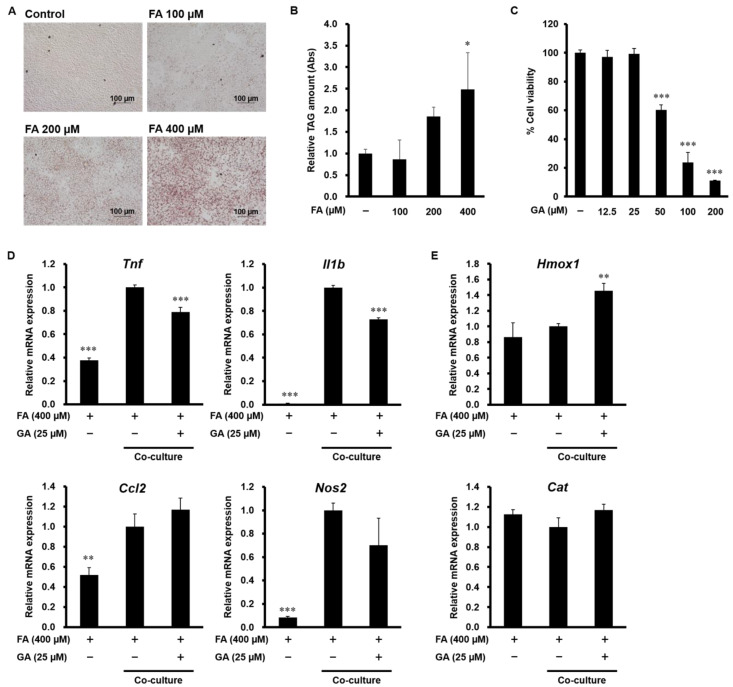
Effect of GA on inflammatory mediator and antioxidant enzyme expression in a co-culture of Hepa 1-6 cells and RAW 264 macrophages. (**A**,**B**) Hepa 1-6 cells were treated with 100–400 µM FA (PA:OA = 1:2) for 24 h. Intracellular triglyceride levels were measured by Oil Red O staining. (**A**) Representative images are shown. (**B**) The extent of steatosis was quantified by the extract in absolute isopropanol. (**C**) Hepa 1-6 cells were treated with 12.5–200 µM GA for 24 h. Cell viability was determined by MTT assay. Data represent mean ± SD, *n* = 3. (* *p* < 0.05, *** *p* < 0.001 compared to control group). (**D**,**E**) Hepa 1-6 cells were treated with 400 µM FA (PA:OA = 1:2) for 24 h, and RAW 264 macrophages were co-cultured on lipid-laden hepatocytes with or without 25 µM GA for 24 h. The mRNA levels of *Tnf*, *Il1b*, *Ccl2*, *Nos2* (**D**), *Hmox1*, and *Cat* (**E**) were determined by real-time PCR. Data represent mean ± SD, *n* = 3. (** *p* < 0.01, *** *p* < 0.001 compared to co-culture group).

To further investigate the effect of GA on chronic inflammation in the liver, experiments using a contact co-culture system of lipid-laden hepatocytes and macrophages were performed. We first evaluated the effect of FA on lipid accumulation in Hepa 1-6 cells by Oil Red O staining. As shown in Figure 5A,B, intracellular triglyceride levels were significantly increased in cells treated with 400 µM FA. MTT assay showed that 12.5 and 25 µM GA did not affect cell viability in Hepa 1-6 cells, whereas high doses of GA (50, 100 and 200 µM) significantly decreased the cell viability (Figure 5C). Therefore, we used 400 µM FA and 25 µM GA treatment in subsequent tests. The mRNA expression of inflammatory mediators, such as TNF-α, IL-1β, MCP-1, and iNOS, was increased in the co-culture compared with the control culture, while treatment with GA significantly attenuated the increase of TNF-α and IL-1β (Figure 5D). Antioxidant enzyme production is important to reduce oxidative stress and inflammation. As shown in Figure 5E, co-culture had no effect on the mRNA expression of HO-1 and catalase, but GA significantly upregulated HO-1 expression.

## 4. Discussion

NAFLD can progress to NASH, which contributes to chronic liver diseases such as cirrhosis and hepatocellular carcinoma [1], and its current treatments have not been widely effective. The interaction of various factors, including hepatic steatosis, apoptosis, and inflammation, is involved in NASH progression [6]. Thus, the inhibition of these pathogenetic factors using dietary-derived phytochemicals would be a therapeutic strategy for targeting NASH and its complications. In previous studies, GA improved hepatic steatosis in high-fat diet-induced NAFLD rats and mice [18,19]. However, the effect of GA on hepatic lipid accumulation, apoptosis, and inflammation, as well as molecular mechanisms, has not been fully elucidated. 

Hepatic steatosis is considered the first stage of NAFLD, which is mostly associated with increased FFA uptake and *de novo* lipogenesis in hepatocytes. In the present study, we demonstrated that GA inhibited PA-induced lipid accumulation in HepG2 cells. Consistent with the inhibition of steatosis, GA suppressed the expression of CD36, FATP2, and ACCα. CD36 and FATP2 are major fatty acid transporters and implicated in the uptake of plasma FFAs and lipid accumulation in hepatocytes [24]. The disruption of hepatocyte-specific CD36 was shown to reduce liver lipid content and improve insulin sensitivity in high-fat diet-induced steatosis mice [25]. Hepatic *de novo* lipogenesis is the biochemical process that synthesizes FAs from acetyl-CoA subunits in the liver, contributing to the pathogenesis of NAFLD [26]. ACCα, the first committed enzyme in the rate-limiting step of *de novo* lipogenesis, catalyzes carboxylation of acetyl-CoA to form malonyl-CoA [27]. In a previous study, liver-specific deletion of ACCα resulted in significant downregulation of FA synthesis and triglyceride accumulation in the liver without affecting fatty acid oxidation and glucose homeostasis [28]. These findings and our results indicate that the suppression of CD36, FATP2, and ACCα by GA may contribute to the attenuation of FFA uptake and lipogenesis, which could improve hepatic steatosis.

SREBP-1c and LXRα are primary transcription factors that regulate FA and triglyceride synthesis. Here, we demonstrated that GA reduced the mRNA expression of SREBP-1c and LXRα. SREBP-1c is activated in animal models of fatty liver, and most lipogenic genes including ACC contain a sterol regulatory element that binds to SREBP-1c [29,30]. SREBP-1c gene silencing decreased ACCα gene expression and lipid deposition in hepatocytes [31]. LXRα directly binds to an LXR-responsive element in the SREPB-1c promoter, thus enhancing its transcriptional activity [32,33]. A previous report suggested that CD36 is also a target gene of LXRα, while LXR agonist-induced hepatic steatosis was largely abolished in CD36 knockout mice [34]. Therefore, our results indicate the involvement of SREBP-1c and LXRα inactivation in the downregulatory effect of GA on CD36, FATP2, and ACCα expression. Through transcriptional regulation by LXRα, SREBP-1c is transcribed as a membrane-bound inactive precursor. In the Golgi apparatus, precursor SREBP-1c is cleaved to mature SREBP-1c that translocates to the nucleus and activates target gene expression [30]. AMPK, a crucial energy sensor of cellular metabolism, inhibits proteolytic processing and transcriptional activity of SREBP-1c by stimulating Ser372 phosphorylation [22,35]. Additionally, AMPK directly attenuates ligand-induced LXR activity, which leads to the suppression of SREBP-1c expression [23]. The present study showed that GA decreased the protein expression of precursor and mature SREBP-1c concomitantly with increased phosphorylation of AMPK. We also determined the role of AMPK on lipid accumulation by using a specific inhibitor. Importantly, the blockage of AMPK signaling significantly reduced GA-induced inhibition of lipid accumulation. These results suggest that the protective effect of GA on lipid accumulation and SREBP-1c-targeted gene expression might be via the suppression of both LXRα-mediated SREBP-1c expression and SREBP-1c cleavage processing through AMPK activation.

Apoptosis is an important mechanism in the progression of NAFLD, and a high rate of hepatocyte apoptosis is observed in NASH patients and animals [36]. Previous studies suggested that lipid-accumulated hepatocytes are susceptible to oxidative stress and subsequent apoptosis, inflammation, and fibrosis, leading to NASH and cirrhosis [8,9]. Consistent with these reports, we showed that simultaneous stimulation of PA and H_2_O_2_ resulted in lower cell viability compared to single stimulation of PA or H_2_O_2_ in HepG2 cells. Once excessive amounts of lipid accumulate in the liver, oxidative stress can oxidize fat deposits, releasing lipid peroxidation products that can activate downstream apoptosis pathways, such as mitogen-activated protein kinase (MAPK) and nuclear factor-kappa B (NF-κB) [37,38]. In particular, p38 MAPK and c-jun N-terminal kinase (JNK) have been reported to activate mitochondrial translocation of Bax and inactivate anti-apoptotic Bcl-2 [39,40,41]. Bax facilitates mitochondrial dysfunction and the release of cytochrome c into the cytosol, which changes caspase-9 to caspase-3 to induce apoptosis. Treatment with a caspase inhibitor reduced apoptosis, fibrosis, and inflammation in the liver of NASH mice [42,43]. In the present study, GA attenuated cell death, the ratio of Bax/Bcl-2 mRNA expression, and caspase 3/7 activity in PA and H_2_O_2_-stimulated HepG2 cells. We previously reported the preventive effect of GA against oxidative stress and MAPK/NF-κB activation [44,45], which might contribute to the anti-apoptotic effect of GA. GA also suppressed the expression of ATF3, a transcription factor that regulates cell proliferation and apoptosis [46]. Previous in vitro studies demonstrated that ATF3 overexpression reduced Bcl-2 expression and induced cell apoptosis [47,48], indicating the involvement of ATF3 suppression in the GA-induced decrease in the Bax/Bcl-2 ratio and subsequent apoptotic changes.

Chronic inflammation is a key step in NASH development, and macrophages play a variety of roles during the inflammatory process including the production of proinflammatory cytokines and chemokines, immune cell recruitment, and dead cell phagocytosis. Itoh et al. [14] reported that the number of hCLS is positively correlated with the extent of liver fibrosis in NASH mice, and the depletion of macrophages inhibited hepatic expression of inflammatory and fibrogenic genes. In this study, we developed an in vitro co-culture system composed of lipid-laden Hepa 1-6 hepatocytes and RAW 264 macrophages. We demonstrated for the first time that the co-culture of Hepa 1-6 and RAW 264 led to marked upregulation of inflammatory genes (TNF-α, IL-1β, MCP-1, and iNOS), suggesting the interaction between these cells. In the co-culture of adipocytes and macrophages, PA, which is a major FFA released from adipocytes, activates MAPK and NF-κB, which mediate inflammatory gene expression in these cells [12,49]. Thus, it will be necessary to investigate whether lipid-laden hepatocytes and macrophages communicate via a paracrine mechanism by using an insert co-culture system. In the co-culture model, we also found that GA reduced inflammatory mediator expression and induced HO-1 expression, which is an antioxidant enzyme involved in the suppression of oxidative stress and inflammation [50]. Concomitant with our previous studies that GA inhibited adverse interaction between 3T3-L1 adipocytes and RAW 264 macrophages [21] and PA-induced inflammation by downregulating MAPK/NF-κB pathway in RAW 264 cells [44], MAPK/NF-κB inactivation may account for the anti-inflammatory effect of GA in the co-culture system. We also showed that GA induced antioxidant enzyme expression via the activation of AMPK in RAW 264 cells [44]. These findings indicate that the changes in inflammatory and antioxidant gene expression by GA might originate in a large part from macrophages in our system. Further research is necessary to clarify the precise molecular mechanisms of GA action on hepatocyte–macrophage interaction.

## 5. Conclusions

In conclusion, GA exerted an inhibitory effect on lipid accumulation through the activation of AMPK in hepatocytes. Our data also showed that GA suppressed hepatocyte apoptosis and inflammatory response in hepatocyte–macrophage crosstalk. These findings provide new perspectives for therapeutic approaches using dietary-derived phytochemicals against NASH and its complications.

## Figures and Tables

**Table 1:** Primer sequences of genes used for real-time PCR.

Species	Gene	Forward Primer (5′ to 3′)	Reverse Primer (5′ to 3′)
Human	*CD36*	CAATTAAAAAGCAAGTTGTCCTCGA	ATCACTTCCTGTGGATTTTGCA
	*SLC27A2*	TCTTGGATGACACAGCAAAAATGT	TCAGAGTTTCAGGGTTTTAGCACTT
	*ACACA*	TCGCTTTGGGGGAAATAAAGTG	ACCACCTACGGATAGACCGC
	*SREBF1*	GAGCCATGGATTGCACTTTC	AGCATAGGGTGGGTCAAATAGG
	*NR1H3*	AGAAGAACAGATCCGCCTGAAG	TTGCCGCTTCAGTTTCTTCA
	*BAX*	GACGAACTGGACAGTAACATGGA	GCAAAGTAGAAAAGGGCGACA
	*BCL2*	GAGTACCTGAACCGGCACCT	GAGACAGCCAGGAGAAATCAAAC
	*ATF3*	TTCTCCCAGCGTTAACACAAAA	AGAGGACCTGCCATCATGCT
	*GAPDH*	TGCACCACCAACTGCTTAGC	GGCATGGACTGTGGTCATGAG
Mouse	*Tnf*	CAAATGGCCTCCCTCTCATC	CTCCAGCTGCTCCTCCACTT
	*Il1b*	TGAGCACCTTCTTTTCCTTCATC	TGTCTAATGGGAACGTCACACAC
	*Ccl2*	ATGCTTCTGGGCCTGCTGT	GGATCATCTTGCTGGTGAATGAG
	*Nos2*	AGGACCACCTCTATCAGGAAGAAA	CAGCTGCTTTTGCAGGATGT
	*Hmox1*	AAGGGTCAGGTGTCCAGAGAAG	GGGAAGTAGAGTGGGGCATAGA
	*Cat*	TATTGCCGTTCGATTCTCCAC	CCCACAAGATCCCAGTTACCA
	*Gapdh*	TGACGTGCCGCCTGGAGAAA	AGTGTAGCCCAAGATGCCCTTCAG
